# Altered differential hemocyte count in 3rd instar larvae of *Drosophila melanogaster* as a response to chronic exposure of Acephate

**DOI:** 10.1515/intox-2015-0013

**Published:** 2015-06

**Authors:** Prem Rajak, Moumita Dutta, Sumedha Roy

**Affiliations:** Cytogenetics Laboratory, Department of Zoology, The University of Burdwan, Burdwan, West Bengal, India

**Keywords:** Acephate, Drosophila melanogaster, plasmatocytes, lamellocytes, crystal cells

## Abstract

Acephate, an organophosphate (OP) pesticide, was used to investigate the effects of its chronic exposure on hemocyte abundance in a non-target dipteran insect *Drosophila melanogaster.* For this purpose, six graded concentrations ranging from 1 to 6 μg/ml were selected, which are below the reported residual values (up to 14 μg/ml) of the chemical. 1^st^ instar larvae were fed with these concentrations up to the 3^rd^ instar stage and accordingly hemolymph smears from these larvae were prepared for differential hemocyte count. Three types of cells are found in *Drosophila* hemolymph, namely, plasmatocytes, lamellocytes and crystal cells. Plasmatocyte count was found to decrease with successive increase in treatment concentrations. Crystal cells showed an increasing trend in their number. Though the number of lamellocytes was very low, a bimodal response was noticed. Lamellocyte number was found to increase with the initial three concentrations, followed by a dose dependent reduction in their number. As hemocytes are directly linked to the immune system of fruit flies, fluctuations in normal titer of these cells may affect insect immunity. Hemocytes share homologies in their origin and mode of action with the immune cells of higher organisms including man. Thus the present findings suggest that immune cells of humans and other organisms may be affected adversely under chronic exposure to Acephate.

## Introduction

Application of pesticides in agricultural fields has been considered as an important tool to repel or kill harmful pests to get a better yield of crops and vegetables. Frequently used pesticides belong mainly to four categories, *i.e.* organophosphates, organochlorines, pyrethroids and carbamates. Each and every category has its own specific way through which it acts on target pests. Organophosphates and carbamates work by blocking the activity of acetyl cholinesterase in the synaptic cleft (Wang *et al*., 2004; Menozzi *et al*., 2004), organochlorines by blocking the activity of either GABA (γ amino butyric acid) gated chloride channels or sodium channels in nerve fibers, resulting in a hyper-excitable state of nerves characterized by tremors and failure in movement coordination (Vijverberg *et al*., 1982). Pyrethroids work by preventing the closure of voltage gated sodium channels in the nerve fibers that lead to repetitive firing of nerve impulses (Soderlund *et al*., 2002).

Besides their main mode of action affecting several factors, pesticides may influence other systems as well. And not only target pests, also non target organisms may get exposed to such chemicals due to consumption of contaminated crops, vegetables and fruits. It is thus topical to investigate the outcomes of pesticide exposure in a non target victim.

Acephate, an organophosphate (OP) foliar pesticide is widely utilized for lepidopteran insects in agricultural fields of tomatoes, lettuce, potatoes and carrots. Like other organophosphates, it blocks mainly the activity of acetylcholine esterase in synaptic clefts. But this chemical has also been reported to shorten the developmental duration in a dipteran insect *Drosophila melanogaster* (Rajak *et al*., 2013). Not only that, its acute exposure (for 12 and 24 hours) was seen to alter differential hemocyte count (Rajak *et al*., 2014). Presence of Acephate residues has been reported in edible parts of some vegetables and fruits (Trevizan *et al*., 2005). Non target consumers feeding on them are thus frequently undergoing chronic exposure to such chemicals. As acute exposure has already been found to affect hemocyte abundance, the authors were curious to investigate the response of hemocytes after chronic exposure to Acephate. Since the European Centre for the Validation of Alternative Methods (ECVAM) has recommended the use of *Drosophila melanogaster* as a model for toxicological assessments and environmental monitoring studies, it was used as a non target insect in the present study.

## Materials and methods

### Test chemical

An organophosphate insecticide Acephate (Rustaf, 75% SP) was used for the present study.

### Experimental organism

Larvae of *Drosophila melanogaster* were used as experimental organism to assess the toxicological impact of Acephate on hemocyte abundance. Larvae were reared in Standard *Drosophila* medium (SDM) following the established laboratory rearing techniques (after Dutta *et al*., 2014; and Podder *et al*., 2012). SDM ingredients included corn meal, sucrose (SRL India), agar agar (Merck, India) and yeast extract powder (Merck, India). Nepagin (Supelco, USA) and Propionic acid (Himedia, India) were added for their antifungal properties.

### Selection of treatment concentrations

Residual values of up to 14 μg/ml of Acephate have been reported in some plants (Fiedler, 1987). Considering this, six graded concentrations of the Acephate ranging from 1 to 6 μg/ml concentrations were selected and accordingly foods containing these chemical concentrations were made for treatment.

### Treatment schedule

1^st^ instar larvae of *D. melanogaster* were fed on food containing different concentrations of the test chemical up to the 3^rd^ larval instar. Control sets were also maintained for comparison.

### Preparation of hemolymph smear

3^rd^ instar larvae were taken out from rearing media and washed thoroughly in Ringer's solution. Hemolymph from three 3^rd^ instar larvae were bled in 10μl of PBS and smear was drawn on a grease-free glass slide. Hemolymph smear was air-dried and then fixed in absolute methanol (Merck, India) for 10–15 min. After fixation, the smear was stained with Giemsa (Qualigens, India) for 20–30 min. Stained slides were washed in distilled water and examined under compound microscope at 400X magnification.

Triplicate slides (each slide made from hemolymph of three 3^rd^ instar larvae) per treatment were prepared and examined. Slides for control group were also counted for comparison purpose.

### Characterization of hemocytes

**Figure 1:**
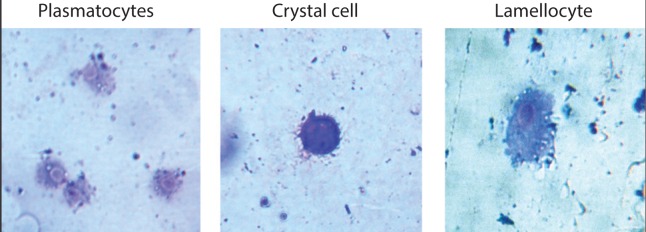
Hemocytes of 3^rd^ instar larvae of *Drosophila melanogaster,* after Giemsa-Rosenfeld staining (400X magnification). Plasmatocytes are large, round cells with irregular margins. These cells are involved in phagocytosis of pathogens and apoptotic bodies. Crystal cells are darkly stained, round and medium sized cells with dark crystals within their cytoplasm. These cells are involved in the melanization process via the prophenoloxidase (proPO) cascade. Lamellocytes are large, oval to elongated cells with prominent dark nuclei. These cells are hardly visible in healthy larvae. Their number increases only during infection. These cells are engaged in encapsulation of large pathogens.

Based upon their morphological features, hemocytes are divided into three categories (Lamaitre & Hoffmann, 2007) (Figure 1).

#### Plasmatocytes

These are the most abundant cells in the circulating hemolymph. They are marked with prominent nuclei and irregular cell margins. Plasmatocytes participate in phagocytosis of pathogens and foreign particles.

#### Crystal cells

These are medium sized, more or less round cells with dark crystals of prophenoloxidase (proPO) inside them. They usually take dark Giemsa stain. Crystal cells are involved in melanin synthesis.

#### Lamellocytes

These are rare but large, flat and oval to elongated cells with prominent nuclei. They are non-phagocytic and their cytoplasmic constituents are relatively sparse. They encapsulate large pathogens which cannot be phagocytosed by plasmatocytes.

### Statistical analysis

All data collected were considered and subsequently mean and standard error of means were calculated using MS Excel 2007. Student’s t test was carried out for analysis of the data to examine the significance in variations in each treatment category compared to their control counterpart. *p*<0.05 was considered statistically significant.

## Results

### Plasmatocyte count

Changes in plasmatocyte count were found to represent a decreasing trend. Untreated larvae showed 91.27±2.13% of plasmatocytes in hemolymph smear followed by 83.35±1.54%, 82.68±3.07% and 77.54±1.81% for treatment concentrations of 1, 2 and 3 μg/ml, respectively.

**Figure 2:**
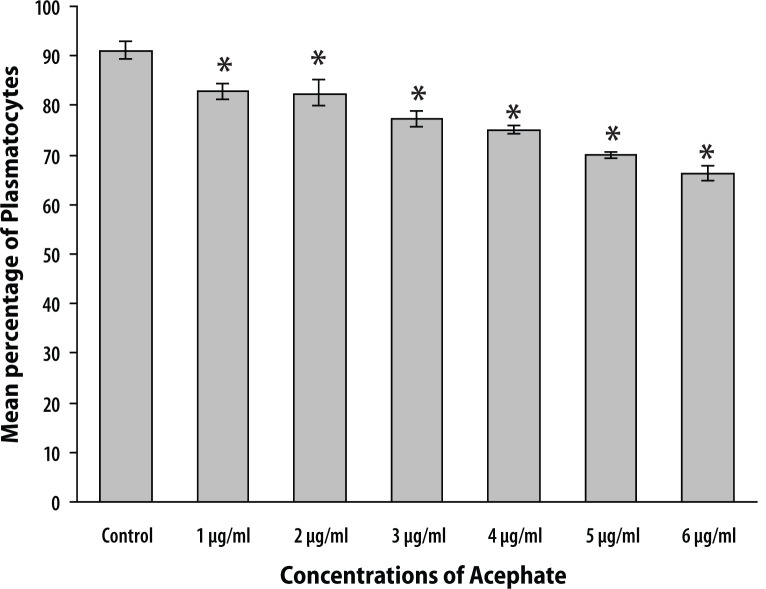
The graph represents reduction in plasmatocyte count with successive increase in treatment concentrations of Acephate. Plasmatocytes in the control set constituted about 91.27±2.13% of total hemocytes, which was reduced up to 66.67±1.44% at higher treatment concentration of 6 μg/ml. Error bars in the graph represent ± standard error. ‘*’ denotes significant reduction in plasmatocyte count when compared with the control group. *p*<0.05 was considered statistically significant.

With further increase in treatment concentrations to 4, 5 and 6 μg/ml, further reduction in the count of these cells was noted representing 75.29±0.79%, 70.43±0.51 and 66.67±1.44% of total hemocytes (Figure 2).

### Crystal cell count

**Figure 3:**
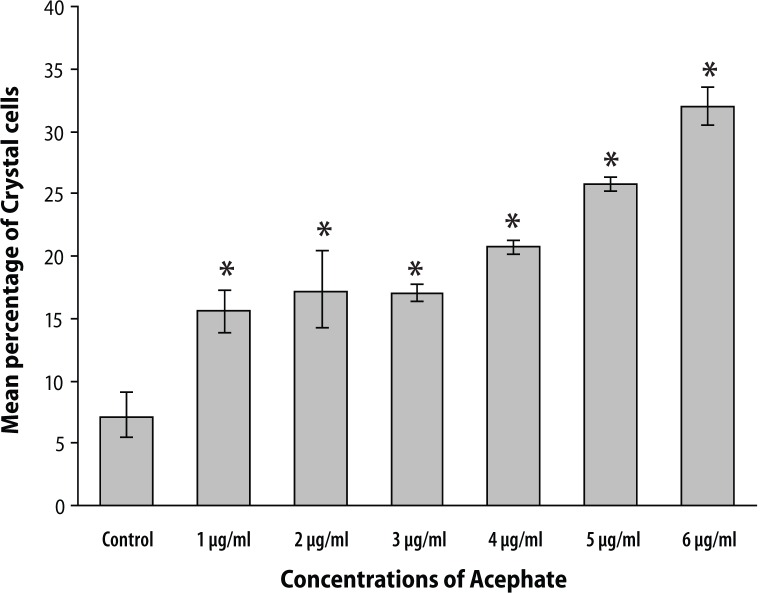
The graph represents a dose dependent increase in crystal cell count with successive increase in Acephate concentrations. Crystal cells in the control set constituted about 7.18±2.09% of total hemocytes which increased up to 32.11±1.58% at higher treatment concentration of 6 μg/ml. Error bars in the graph represent ± standard error. ‘*’ denotes significant increase in crystal cell count when compared with the control group. *p*<0.05 was considered statistically significant.

In contrast to plasmatocyte count, crystal cells exhibited an increasing trend in relative abundance with successive increase in treatment concentrations. Control sets revealed about 7.18±2.09% crystal cells. With graded chemical concentrations of 1, 2 and 3 μg/ml, a respective increase in crystal cell count of 15.75±1.63%, 17.25±3.34% and 17.16±0.79% was recorded. Larvae reared in 4, 5 and 6 μg/ml concentrations constituted about 20.87±0.57%, 25.84±0.67% and 32.11±1.58% of total hemocytes (Figure 3).

### Lamellocyte count

**Figure 4:**
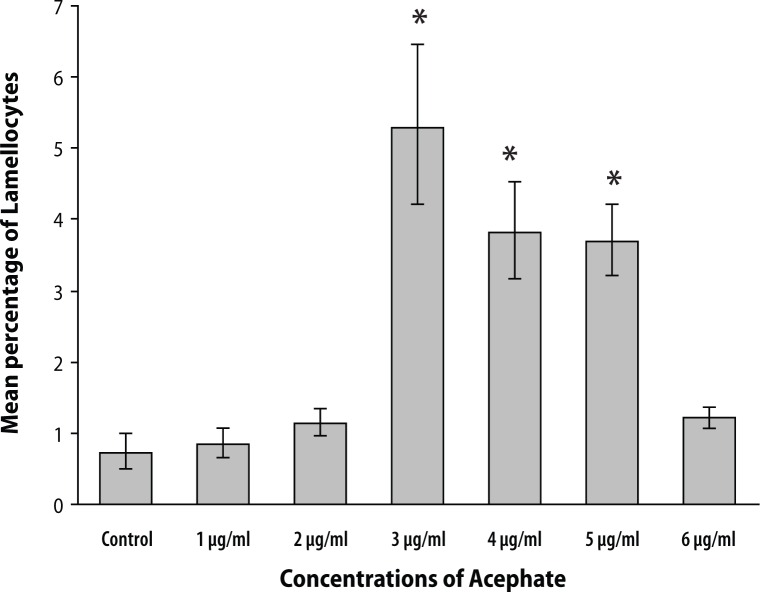
The graph represents changes in lamellocyte count in response to chemical insult. Lamellocyte number was found to increase in case of the initial three chemical concentrations achieving maximum number at 3 μg/ml treatment concentration. Beyond 3 μg/ml, the lamellocyte count was noticed to decrease and reached 1.22±0.14% at 6 μg/ml treatment concentration. Error bars in the graph represent ± standard error. ‘*’ denotes significant variation in lamellocyte count when compared with the control group. *p*<0.05 was considered statistically significant.

Though lamellocyte numbers were very few in hemolymph smears, fluctuations in their relative abundance were observed. Lamellocytes in the control set constituted about 0.73±0.28% of total hemocytes. Chronic exposure for several days to three initial concentrations (1, 2 and 3 μg/ml) of Acephate resulted in increased respective lamellocyte count of 0.86±0.23%, 1.15±0.21 and 5.29±1.17%. Interestingly, with further increase in treatment concentrations to 4, 5 and 6 μg/ml, the relative abundance of these cells was found to decrease to 3.83±0.71%, 3.71±0.52% and 1.22±0.14%, respectively (Figures 4).

## Discussion

The present work revealed the effect of chronic exposure to Acephate on relative abundance of hemocytes in the hemolymph of 3^rd^ instar larvae of *D. melanogaster.* Plasmatocyte abundance was found to decrease under chemical stress. Comparable results were reported by Qamar and Jamal (2009) with the same insecticide Acephate leading to reduced plasmatocyte count in the insect *Dysdercus cingulatus.* Other organophosphates like methylparathion and monocrotophos were also noticed to decrease plasmatocyte abundance in *Rhynocoris kumarii* (George & Ambrose, 2004). Reduction in the number of these cells might be a product of increased apoptosis, as Acephate is considered as an apoptotic inducer (Tripathi *et al*., 2007). Some other organophosphates like profenofos and methyl parathion are also known to reduce the rate of mitosis (Ganguly *et al*., 2010), therefore proliferation of plasmatocyte precursor cells during hematopoiesis might be targeted by Acephate resulting in decreased cell number.

Crystal cell number showed an increasing trend in abundance with successive increase in treatment concentrations. Melanization has been considered as an important tool to minimize damage from physical, chemical and pathogenic stresses (Hamilton & Gomez, 2002). Crystal cells possess dark crystals of prophenoloxidase (proPO) in their cytoplasm which gives them their characteristic appearance. Under stressed conditions, the serine protease cascade may become activated, ultimately cleaving proPO to their active form of phenoloxidase (PO). PO in turn oxidizes phenols into quinones. Quinones then polymerize to produce melanin (Meister & Marie 2003). Melanin is used to combat stress conditions. As crystal cells are directly linked with the melanization process, their proliferation increases in response to a chemical insult to synthesize a more abundant amount of melanin in order to minimize the damage caused by chemical stress. The insecticide resistant strain of *Culex pipiens* was found to possess higher serine protease activity than non resistant strains (Gong *et al*., 2005; Yang *et al*., 2008). This higher activity might be associated with a detoxifying mechanism for insecticides. An increased crystal cell number provides a clue regarding the involvement of serine protease, proPO and melanin in the detoxification mechanism of the current insecticide.

A similar kind of response for plasmatocytes and crystal cells was previously reported by Rajak *et al*. (2014) where the same chemical reduced the plasmatocyte count followed by an increased number of crystal cells under acute exposure to Acephate for a period of 12 and 24 hours. Yet interestingly, the response of lamellocytes was different from the present study. Under acute exposure to Acephate, the lamellocyte count was reduced in a dose dependent manner, but under chronic exposure, lamellocytes showed a bimodal response in which the initial three concentrations used led to an increase in the number of lamellocytes, followed by reduction in the abundance with the last three higher concentrations.

Lamellocyte number is known to increase under pathogenic stress (Evans *et al*., 2003) and it works by inactivating the foreign particles including parasites through encapsulation process (Meister, 2004). Chemical particles consumed into the gut might be absorbed into the hemolymph, which stimulates the proliferation of lamellocytes to encircle these particles for the detoxification process. The last three concentrations of the test chemical resulted in diminished lamellocyte number, similarly to that of plasmatocytes. This might be due to stimulation of apoptosis or mitotic failure of lamellocyte precursor cells at the given higher concentrations.

## Conclusion

The present study explored that, whether Acephate at selected concentrations interferes with normal hemocyte number of 3^rd^ instar larvae of the fruit fly. The findings indicate the involvement of apoptotic induction, mitotic failure and the effect on the hematopoietic mechanism of this insect. The hematopoietic system of *Drosophila* shares a high degree of homology with that of human beings, hence the study points toward the undesired alarming outcome of irrational use of such chemicals in the environment.
